# The Process of Decomposition

**Published:** 1872-04

**Authors:** 


*The Process of Decomposition.* An abstract from Caspar's Handbuch der Gerichtlichen Medicin. Berlin, 1871.

In No. 10 of *The Clinic* we endeavored to lay before its readers, concisely, the views of Caspar on the signs of death, as developed in his exhaustive work on medical jurisprudence, leaving the consideration of the process of decomposition, with reference to the time of death, for a separate article.

We will now turn our attention to this subject. Caspar says it is surrounded with many difficulties. It is not easy to describe, by mere words, the various changes in color and consistency of organs which the corpse undergoes, and in view of the many agents that affect the process of decomposition anything like a general rule can be laid down only with great caution. He thinks it is scarcely an exaggeration when Orfila says that "it is beyond the powers of man" to determine the time of death in corpses that have undergone decomposition. And yet, Devergie says of his subordinates in the Paris Morgue, and Caspar has noticed the fact himself in Berlin, that men without any culture whatever, acquire, by mere routine, a judgment in this matter, which, in general, is quite correct; whence our author argues it must be possible to do it still more accurately by employing scientific means. Whilst appreciating the labors of Orfila, Lesueur, Guntz and Devergie,* he censures them for devoting too much attention to trifling details, making their works practically worthless for the medical jurist.

INTERIOR CONDITIONS OF DECOMPOSITION.

The causes which modify the process of decomposition, by either hastening or retarding it, so that the corpse of A may in from 24 to 36 hours present the same appearance as the corpse of B in from three to four weeks, must be sought in the individual, or *externally*. As a matter of course, decomposition itself is possible only through exterior influences. Fresh flesh, hermetically sealed, does not decompose.

Individually, the process of decomposition is modified—

1. By *age*. Caspar admits what all authors maintain, that *neo-nati*, *cet. par.*, undergo decomposition more rapidly than other corpses. Still, we have to take into consideration the fact not noticed by any other writer, that they are nearly all found naked immediately after birth, or at most, enveloped in a few rags, having been exposed, or thrown into the water, into vaults, or hidden in manure or rubbish; whilst naked corpses of a greater age are almost exclusively only those of persons drowned. But the influence of clothing in retarding the process of decomposition is very essential.

2. *Sex*, according to Caspar, has no influence. But he has noticed that the corpses of women, who died immediately after parturition, decomposed very rapidly, no matter what was the cause of death.

3. The *constitution* has a very decided influence. Fat, spongy and lymphatic bodies undergo decomposition far more rapidly than bodies lean and dry, because an abundance of liquids favors the process. In the corpses of the aged this process is retarded, probably, because they are generally lean and dry.

4. *The mode of death* has a marked effect on the process of decomposition. It occurs much later after the sudden death of healthy persons than in persons dead of diseases which exhaust

* Orfila u. Lesueur, Handb. z. Gebr. f. Gerichtl. Ausgrabungen, Leipzig, 1832–35.
Guntz, der Leichnam des Nengeborenen, Leipzig, 1827.
Devergie I, p. 88—253.

the system; such as typhus, dropsy following organic lesions, or tuberculosis. Mutilated bodies, and those of persons killed by repeated acts of violence, by mechanical force, on railroads, etc., decompose rapidly. Excepted from this rule are the bodies of persons that were killed by the falling of walls; the stones, rubbish, or sand with which they remained covered, preventing a direct contact with the air. Persons suffocated by smoke, carbonic oxide gas, or sulphuretted hydrogen, decompose rapidly. After death from narcotic poisons, decomposition is accelerated. This is much less the case after other poisons, and especially phosphorus. Where the blood was poisoned by alcohol, i. e., in cases of drunkards who died of apoplexy, in a state of intoxication, Caspar observed that the corpses retained their freshness for a remarkably long time.

It is worthy of notice, finally, that after poisoning by sulphuric acid the process of decomposition is decidedly retarded, probably because the acid prevents the formation of ammonia, or continues to neutralize the ammonia formed by decomposition. After poisoning by arsenic decomposition occurs according to the ordinary laws, but it is well known that it is interrupted in its course by the process of mummification, of which more hereafter.

We ought to remember, however, that, although the foregoing statements are generally correct, there must be still other causes, as yet unknown, which hasten or retard decomposition.

As cases in point, the following are cited by Caspar:

On March 18th, 1848, two men suffered the same death, by gunshot wounds, at the same time, on the barricades in Berlin. Nevertheless, the signs of decomposition were dissimilar. At another time, a man and a woman, both aged between 50 and 60 years, died the same night from suffocation by carbonic oxide gas. Four days after, the man's corpse was quite green on the abdomen and back, whilst that of the woman, though remarkably fat, was, internally as well as externally, quite fresh.

### EXTERIOR CONDITIONS OF DECOMPOSITION.

The exterior conditions have a more decided effect on decomposition than the interior. They are air, water and heat.

*a. Atmospheric air.* Everything that favors or hinders its access to dead animal (as well as vegetable) substances, hastens or retards the process of decomposition. Helmholz* has shown that no decomposition takes place in flesh that is in contact with water cleared by boiling and air conducted through red-hot tubes, until all organic germs are destroyed. The oxygen of the atmospheric air is, consequently, the chief condition of decomposition. Corpses remaining in the open air decompose much more rapidly than

* Erdmann u. Marchner, *Journal d. Pract. Chemi.*, Bd. 31, s. 420.

those that are buried, and even than those lying in water. Decomposition sets in sooner in naked or lightly clothed corpses than in those that are completely dressed, especially if the clothes are tight and but little permeable.

The access of air may be obstructed or favored by the composition of the earth. If it is light and porous, decomposition will commence sooner than if it is moist and dense. But here a great deal must be ascribed to the action of water, because light earth (sand) is at the same time dry, whilst moist earth (clay) is naturally damp. So, too, corpses decompose sooner in shallow graves than in those of great depth. It is further important to consider the cover that surrounds the corpses in the earth, which is well shown by Orfila. Every body knows how soon coffins of common pine are destroyed, and, with them, the corpses; whilst the great men of former times, buried in coffins of hard wood, tin or stone, were preserved for an uncommonly long period. On the contrary, corpses that were buried in an entirely nude state decompose very rapidly.

*b. Water.* Without water and watery vapor no decomposition takes place. But the water of the corpse itself is sufficient. It gradually evaporates, and, eventually, bursts the walls of the abdomen, of the thorax, and, finally, even the bones of the skull, and the corpse macerates in its own liquids. Before this time, however, maggots and larvæ make their appearance on the surface, at first on the eyelids, ears, vulva and groins, until their number is increased to myriads, and the complete and total destruction of all the soft parts follows.

But in proportion as water without has access to the corpse, decomposition will be more rapid, and *vice versa.*

*c. Heat.* A high degree of heat, alone, causes the liquids of the body to evaporate, and favors the very opposite of the process of decomposition, i. e., the drying up if not roasting of the corpse, as we see it in combustion. Ordinary heat, however, when combined with air and humidity, favors decomposition. It is well known that a corpse will decompose more rapidly in summer than in winter. Bodies that at a summer temperature of 16 to 20° R. are still in a good state of preservation, may, in the course of 24 hours, become entirely unfit for dissection, whilst in water, at a temperature of from 5 to 8° R. this will not be the case for 10 or 12 days. The difference of temperature is exceedingly remarkable in the case of water. If the corpse freezes in water (or humid earth) it will be preserved for a long time, even for thousands of years, as is shown by the mammoth, exhumed in Siberia, and now to be seen in the Museum of the University at Moscow, though the remains of the tissues are partly saponified. In winter, a corpse taken from water showing a temperature of from 2 to 6° R. 10 to 12 days after death, may still be so well preserved as to enable an examiner to demonstrate the signs of death from suffocation, which in summer, at a temperature of from 18 to 20° R. is often impossible after 5 to 7 days; and here, another circumstance must be considered. It is well known, that the temperature of the water is lower, under its surface, than on it and in the superior strata, because the rays of the sun strike only these. Decomposition will, therefore, be accelerated when the corpse is on the surface, and retarded when it remained at the bottom. Another point that must not be overlooked is this, that a corpse, taken from the water and exposed to the air, will decompose with exceeding rapidity. The progress will be greater in one day than it would have been in three or four days, had the corpse remained in the water. So, too, a higher or lower degree of the temperature of the earth affects decomposition. Corpses buried in shallow graves, will, therefore (as also for the reason given above under *atmospheric air*) decompose more rapidly than those in deep graves.

COMPARISON OF THE SIGNS OF DECOMPOSITION ACCORDING TO THE MEDIA.

Caspar says it is perplexing for the practitioner to divide the process of decomposition into stages according to the different media, as has been done by Orfila, Devergie and Guntz, and he thinks it is superfluous, because this process is, in all cases, one and the same from the first moment to the last, modified only by the media and all the various conditions heretofore enumerated. It appears, to him, more judicious to set up a general criterion regarding all three media, i. e., air, water and earth, leaving the effect of the other conditions to be considered in each given case. Aware of the difficulty of setting up such criterion, yet from his large experience he does not believe he departs from the truth in announcing the following doctrine: *the average degree of temperature being about equal, a corpse exposed to the open air for one week (or month), exhibits the same degree of decomposition as a corpse that remained in water two weeks (or months) and one that was buried in the earth in the usual way, for eight weeks (or months).*

The same degree of decomposition will therefore, *cet. par.*, be shown by three corpses of which *a* has been exposed to the air, e. g., on the ground, for a month; *b* was drowned two months ago; and *c* has been dead and buried in an ordinary coffin for eight months. A due regard for this rule and a proper intimation of the circumstances of each specific case, will avoid *essential* errors.

SUCCESSIVE CHANGES OF THE CORPSE, EXTERNALLY.

1. The first sign is the well known greenish color of the walls of the abdomen, on the appearance of which the odor of decomposition is noticed. It occurs in from twenty-four to thirty-six hours. (The exception in the case of persons found drowned will be referred to hereafter.)

2. In the same time, the eye-balls soften, and yield to the pressure of the finger.

3. In from three to five days after death this green color becomes more marked and covers the entire abdomen, including the exterior sexual organs. In the latter, it assumes, in both sexes, a rather brownish-green, dirty appearance from the start. In a great many corpses, especially in cases of suffocation, sanguineous, frothy liquids, with air-bubbles, ooze from nostrils and mouth. At the same time, green spots appear, with great topical irregularity, on other places, especially on the back, the lower extremities, the neck, and lateral portions of the thorax.

4. In from eight to twelve days after death this discoloration, with which the odor keeps even pace, has covered the entire body and become darker. In some places, especially on the face and on the entire neck down to the breast, the color, even at this stage, is reddish-green, the decomposed blood having extravasated into the cellular tissue. Putrid gases are developed, and inflate the abdomen. These are (but not in all cases) combustible gases, sulphuretted and phosphuretted hydrogen. On making an incision in the walls of the abdomen, the gas which escapes, will, on lighting it, burn for a considerable time, with a small flame. This takes place, almost without exception, with the gas proceeding from the scrotum. The cornea has become concave, but the color of the eyes may still be recognized, whilst it is no longer possible, in all cases, to determine whether the pupils of the immature fœtus are open or not. The *sphincter ani* is open. In some places of the corpse, especially on the extremities, on the neck and breast, cutaneous veins of a dirty red may be seen through patches of skin of a lighter color. The nails are still firm.

5. In from 14 to 20 days after death, the color of decomposition has everywhere become green, like the color of the frog, and bloodred-brown. The cuticle, in some places, is raised in blisters of the size of a walnut; in others, patches as large as a plate, and still larger, are entirely detached. Innumerable maggots cover the body, seeking especially the folds of the skin and the natural cavities. The evolution of gas has increased to such an extent, that the abdomen looks like a large ball, the thorax is artificially raised, and the entire cellular tissue presents the appearance of inflation. The corpse resembles that of a giant. The features can no longer be distinguished, and even intimate friends find it difficult to recognize the body; for the lids, the lips, the nose and the cheeks appearing much swelled, the physiognomy is naturally entirely different. The color of the eyes can no longer be distinguished, because the eye-ball (iris and pupil being obliterated) shows in all such corpses, without exception, a uniform dirty-red color in the entire extent of the sclerotica. In men, the penis has become enormously large, and the scrotum may reach the size of a child's head. The nails are detached with their roots, and may be easily pulled from the limbs. The scalp comes off readily.

Temperature is an important condition in bringing about this high degree of decomposition. Taking the extremes of temperature, it may be said, that 16 to 20° R. in summer, will have the same effect in from 8 to 10 days as 0 to 8° R. in winter in from 20 to 30 days. We mentioned that at this stage of decomposition the corpse is covered with maggots. But where it was exposed to the open air or had lain in the water, it is not uncommon to find that it has been a prey to other animals, especially rats, but also dogs, cats, birds of prey, foxes and wolves. The evidences of such voracity are found on the chest and abdomen which frequently have been opened; and on the extremities, which, in many places, appear as if dissected down to the bones. A little attention will prevent us from confounding such lesions with *traumatic effects.* Finding a corpse in the condition just described, we may affirm with some degree of certainty, that the individual has been dead *at least* as long as above stated, but not that it could not have been dead longer, for this stage of decomposition differing thus from former stages, continues, generally, many weeks, even a few months, and but very gradually proceeds to the following stage: *Corpses in a state of putrescence, of a green color, inflated and excoriated, found one month after death, cannot well, cet. par., be distinguished from similar corpses found in from three to five months after death.*

6. The stage of putrid colliquation begins in from four to six months after death; in corpses lying in warm and humid media, even sooner. The constant evolution of gas has opened the cavities of the chest and abdomen. Often the bones of the skull, too, have yielded in the sutures and the brain has escaped. All the soft parts are in a state of pap-like solution, or have been partly dissolved and consumed. Entire bones, especially those of the skull and extremities, are bare. Those of the extremities are often, even at this stage, detached at the joints; the fasciæ and ligaments being destroyed. No trace of the physiognomy can be recognized. The existence of mammæ can no longer be determined, and since the external sexual organs have also disappeared, the sex of the individual can be known from the exterior *habitus* only in case the hair of the pudenda is still visible, which is often the case. As is well known, in a female, this hair is strictly confined to the *mons veneris*, in the male it extends to the *umbilicus*. According to Schulze, exceptions to this rule are not rare.* It is also still possible to determine the sex by searching for a *uterus*.

[To be concluded.]

* Jenaische Zeitschrift, Bd. iv. Heft 2, S. 312.

*Constriction of the Penis by a Wedding Ring.* By M. DUMAREST, M.D., (Lyons). Interne des Hopitaux. From the Lyons Medicale.

G——, married, aged 59. Entry Sept. 27, 1871. Eight days before entry, with a design at least original, he introduced his penis into his wedding ring. The ring was imbedded behind the glans and prepuce. The prepuce was in the condition of paraphymosis and the constriction of the ring had produced an ulceration of unequal depth; it was very deep on the upper surface of the penis, superficial over the urethra on the under surface. Micturition was difficult but still possible.

The ring was divided in three segments, subsequent cicatrization was rapid; two days afterwards he left the hospital almost cured.

In this patient notwithstanding the violence and long duration of the constriction, though there was ulceration, there was no gangrene. Gangrene of the penis from mechanical cause is very rare, whether the cause be artificial constriction or by paraphymosis. In truth ulcerations from a ligature of the penis, ulceration not only of the dorsum but of the much more resistant under surface, ulceration so extensive as to produce urethral fistula may exist and yet gangrene will not ensue. M. Laroyenne, notably, has seen two cases of ulceration of this kind in children fearful of punishment who tied the penis to avoid wetting the bed. Our patient is a proof that sphacelus does not present as easily as most of the authors claim.

It is useless to add that fatal mortification of the penis may ensue if the constriction be of long duration and excessively violent. As to paraphymosis it is generally admitted that it may be easily complicated with gangrene; far from being true, this complication is extremely rare. M. Laroyenne has never observed it. In all cases it easily produces ulcerations of the prepuce and furrow. This ulceration may cause the most serious dangers on the under surface, if it involve the urethal canal. It may be the point of departure of an urethral fistula, or indeed of an immediate urinary infiltration, or, finally, of a future stricture. But the circulation is extremely rarely sufficiently arrested at the outset to induce, by the sole fact of constriction, mortification of the glans or the cavernous body. M. Demarquay* and many others with him are wrong, then, in admitting its frequency, with either phymosis or paraphymosis.

Cases of ligation or constriction of the penis by foreign bodies are not rare. A few are encountered everywhere. We cite a few as follows:

* Archives de Med., 1870.

M. Demarquay has reported two; the first was published by M. Leteinturier, of Havre. The introduction of the penis into a ring was followed by mortification of the skin of the entire penis and the anterior part of the scrotum. The patient was a peasant who had used this token of love from his mistress in this way as a charm.

The second was that of Bourgeois* who did not hesitate to remove a portion of the corona glandis to facilitate the detachment of the constricting ring. It was a radical procedure: the patient was released from it with a few eschars upon the prepuce and the dorsum penis from which he recovered in two months.

Nat. Guillot reported the case of a baker who in the first months of marriage permitted his wife to pass over the penis the bond which she had carried on her finger. This ring was gold, and a pharmaceutist conceived the ingenious idea which released the patient. He dissolved it in a bath of mercury.

M. Guibout, in 1868, communicated to the Societe des Hopitaux the case of a man of more than 60 years of age who had introduced his penis up to the root in *seven* rings of copper, very small and very strong, "to procure," as he said, "a few moments of pleasure." These rings remained in place eleven hours. The penis had become hard, cyanosed, extremely turgid, and of gangrenous hue. After a thousand efforts the seven rings were successively divided. In eight days every local trace had disappeared.

Two well known cases we finally cite: the one of a soldier in whom Larrey found the penis fastened in the socket of his bayonet, and the other that of the bather found suspended by the penis from the cock of his bath tub.—*The Clinic.*

* Mem. de l'Acad. Roy. de Chirurg., vol. I.

